# Predictive Value of Triglyceride-Glucose Index for All-Cause and Cardiovascular Mortality in Patients With Diabetes Mellitus: A Retrospective Study: TyG Index and Mortality in Diabetes

**DOI:** 10.1155/2024/6417205

**Published:** 2024-10-23

**Authors:** Xiaoxuan Feng, Yishou Deng, Chaolei Chen, Xiaocong Liu, Yuqing Huang, Yingqing Feng

**Affiliations:** ^1^Institute of Hypertension, Guangdong Provincial People's Hospital (Guangdong Academy of Medical Sciences), Southern Medical University, Guangzhou 510080, China; ^2^Guangdong Cardiovascular Institute, Guangdong Provincial People's Hospital, Guangdong Academy of Medical Sciences, Guangzhou 510080, China; ^3^Department of Rehabilitation, The First Affiliated Hospital of Jinan University, Guangzhou 510630, China

**Keywords:** all-cause mortality, cardiovascular mortality, diabetes mellitus, insulin resistance, triglyceride-glucose index

## Abstract

**Objective:** To determine the associations between triglyceride-glucose (TyG) index and mortality from all causes and cardiovascular causes in diabetic population.

**Methods:** 3349 participants with diabetes mellitus (DM) from the 1999–2014 National Health and Nutrition Examination Surveys (NHANES), aged 18–85 years were included and grouped based on the TyG index in quintiles. Mortality was followed up through December 31^th^, 2015. Cox proportional hazards models were used to assess the hazard ratios (HRs) and 95% confidence intervals (CIs). We clarified the shape of association between TyG index and mortality using restricted cubic splines and piecewise linear regression.

**Results:** After a median follow-up period of 82 months, 800 (23.9%) deaths occurred, of which 190 (5.7%) were due to cardiovascular causes. Participants in the top quintile had higher risks of all-cause mortality (HR, 1.38; 95% CI, 1.04–1.48) and cardiovascular mortality (HR, 2.43; 95% CI, 1.32–4.45) than those in the lowest quintile. TyG index and all-cause mortality had a J-shaped relationship with a threshold value of 9.32, while TyG index and cardiovascular mortality had a reversed L-shaped relationship with a threshold value of 9.37. Higher TyG index was associated with increased risks of all-cause mortality (per SD increment, HR, 1.52; 95% CI, 1.27–1.82) and cardiovascular mortality (per SD increment, HR, 2.17; 95% CI, 1.54–3.04) when above the threshold values. The sensitivity analyses demonstrated similar findings.

**Conclusions:** TyG index in diabetic patients was nonlinearly correlated with mortality risks, potentially predicting all-cause and cardiovascular mortality.

## 1. Introduction

Diabetes mellitus (DM) is a globally social and public problem with high prevalence, disability rate, fatality rate, and relatively low early diagnosis rate, resulting in a heavy global medical and economic burden [1]. DM is characterized by the failure to compensate for insulin resistance (IR), which is tightly correlated with an increased risk of mortality [2]. The mechanism of IR is insulin-mediated glucose metabolic homeostasis sensitivity and responsiveness attenuation, as well as suppression of hepatic glycogen heteroplasia, which can result in hyperglycemia and dyslipidemia [2]. IR also increases the prevalence of cardiovascular diseases (CVD) and complications among the diabetics [3, 4]. As a result, diabetic population must be evaluated for IR.

Traditional methods for validating IR include hyperinsulinemic euglycemic clamp (HEC) and homeostasis model assessment-insulin resistance (HOMA-IR). Nevertheless, both HEC and HOMA-IR are ambitious, expensive in clinical practice and difficult to apply to some patients, such as those with ineffective glycemic control and severe *β*-cell dysfunction [5, 6]. Triglyceride-glucose (TyG) index, composed of fasting blood glucose (FBG) and triglycerides (TG), has been proposed as a candidate for a simple, convenient and low-cost alternative indicator of IR [7]. Numerous studies manifested that TyG index is intimately related to HEC and HOMA-IR [7]. Both DM and the TyG index were found to be separate predictors of long-term major adverse cardiovascular events (MACE) in patients with a high risk of CVD [8]. TyG index has been widely studied for the correlation between DM and outcomes including micro- and macroangiopathies [9], arterial stiffness [10], coronary artery disease [11], diabetic kidney disease [12], as well as the occurrences of major adverse cardiovascular and cerebral events [13]. However, there is presently precious little research with regard to the association between TyG index and mortality in diabetic individuals. Therefore, we conducted a retrospective cohort study to explore the relationship between TyG index and mortality from all causes and cardiovascular causes in diabetic population.

## 2. Materials and Methods

### 2.1. Data Source and Study Population

The population data in the present study came from participants in the NHANES administered by the Centers for Disease Control and Prevention, is intended to assess the health status of US citizens residing in 50 states and the District of Columbia from 1999 to 2014 [14, 15]. Data collection takes place year-round in 30 sampling sites throughout the US per 2-year cycle [14, 15]. The final follow-up date was December 31^st^, 2015. The study protocol was approved by the institutional review committee (National Center for Health Statistics Ethics Review Board Approval Protocol Number: #98-12, #2005-06, #2011-17) of the Centers for Disease Control and Prevention (CDC), and written informed consents were attained from participants [16]. More details of NHANES are available for online access [17]. Data for analysis was taken from NHANES (1999-2014) with 82,091 participants in total. Accordingly, we included 47,356 participants aged 18–85 years old, regardless of gender. Subjects without blood lipids data (*n* = 26,780), FBG data (*n* = 50), follow-up data (*n* = 22), or baseline without DM (*n* = 17,149) were excluded. Participants with FBG <126 mg/dL, no self-reported DM history, hemoglobin A1c (HbA1c) < 6.5%, and not using hypoglycemic drugs at baseline had no DM [18]. The final analytical population comprised 3349 individuals with DM (Figure S1).

### 2.2. Measurement of Blood Samples

Serum samples were obtained from a peripheral vein while the subjects were fasting. All participants performed a fasting glucose test in the morning session who were required to be after fasting for at least 9 h. Then the FBG level was measured by the hexokinase method [19]. TG and total cholesterol (TC) were tested using enzymatic assays, whereas high-density lipoprotein cholesterol (HDL-C) was evaluated using either a heparin-manganese precipitation method or a direct immunoassay methodology. The Friedewald formula was used to compute low-density lipoprotein cholesterol (LDL-C). TyG was determined using the formula: ln [fasting TG (mg/dL) ∗ FBG (mg/dL)/2] [7].

### 2.3. Outcomes Definition

In the present study, we incorporated mortality cases resulting from CVD and all causes. We utilized the public-use linkage file for NHANES and mortality as of December 31^st^, 2015 to ascertain data outcomes, which were correlated with the National Center for Health Statistics (NCHS) with the National Death Index (NDI) through a probability matching algorithm. During follow-up, participants who did not comply with death certificates were considered alive. We calculated the follow-up time from the interview date to censored date (alive or dead). All-cause mortality contained all cases resulting in death. Cardiovascular deaths were identified using International Classification of Diseases, 10th Clinical Modification (ICD-10) system codes (I00–I09, I11, I13, I20–I51, I60–I69).

### 2.4. Covariates

Height, weight, systolic blood pressure (SBP), and diastolic blood pressure (DBP) were collected by physical examinations. Demographic data on age, sex, race (white or non-white), smoking (current or ever), self-reported medical history including CVD and DM, and medication use (antiplatelet medications, lipid-lowering drugs, hypoglycemic agents, and antihypertensive drugs) were gathered by normalized questionnaires. CVD history included coronary artery disease, angina, heart attack, or stroke. Body mass index (BMI) was computed by dividing weight in kilograms by height in meters squared. Estimating glomerular filtration rate (eGFR) was attained by Modification of Diet in Renal Disease (MDRD) equation formula [20]. Renal failure was defined as eGFR < 30 mg/min/1.73 m^2^ [21]. HbA1c was quantitatively measured by the Tosoh Automated Glycohemoglobin Analyzer HLC-723G8. Hypertension was defined as SBP ≥ 140 mmHg, DBP ≥ 90 mmHg, reported the use of hypertensive drugs, or self-reported history of hypertension [22]. The presence of heart failure and cancer was ascertained by asking participants if a doctor or healthcare provider had ever diagnosed them with congestive heart failure and cancer/malignancy. For these questions and other self-reported data, any answer other than “yes” was assumed to be “no.” [23].

### 2.5. Statistical Analyses

Subjects were classified by TyG index in quintiles (Q1: ≤ 8.63, Q2: 8.63–9.00, Q3: 9.01–9.35, Q4: 9.36–9.82, Q5: ≥ 9.83). Variables of a categorical nature were represented as frequencies and percentages. Continuous variables were displayed as mean value with standard deviations (SD) after applying the Kolmogorov–Smirnov test to verify normality distribution. Baseline characteristics among groups were compared by chi-square test, one-way ANOVA or Kruskal–Wallis H-test when appropriate. We used Kaplan–Meier curves and the Log-rank test to perform survival analysis. Three models built by multivariate Cox regression were used to estimate adjusted hazard ratios (HRs) with 95% confidence intervals (CIs) of TyG index for mortality from all causes and cardiovascular causes. Model 1 consisted just of the TyG index. Model 2 was adjusted for age, gender, as well as race. Model 3 was additionally adjusted for smoking, BMI, SBP, eGFR, TC, HDL-C, cardiovascular disease, hypertension, antiplatelet medications, hypoglycemic agents, lipid-lowering drugs, and antihypertensive drugs. The nonlinear relationship between TyG index and mortality was then identified by Cox restricted cubic spline models. Assuming that the nonlinear relationship existed, we utilized two-piecewise linear regression models to illustrate how the association differed around the threshold points, which chose the one with the highest likelihood value after computing all possibilities. Logarithmic likelihood ratio test was carried out in order to examine the disparities in the relationships discovered by employing one-line linear regression models as opposed to two-piecewise linear regression models. For the sake of investigating the sources of potential heterogeneity, subgroup analyses were conducted on age (< 65 versus ≥ 65 years), gender (male versus female), race (white versus non-white), and BMI (< 25 versus ≥ 25 kg/m^2^) stratification. Sensitivity analyses was conducted by (1) excluding participants with cancer, heart failure, or renal failure and (2) imputing the missing total energy variable with the median value and including the total energy variable into the analysis. All analyses were conducted using R version 3.6.3 (R Foundation for Statistical Computing, Vienna, Austria). *p* < 0.05 was regarded as statistically significant.

## 3. Results

### 3.1. Baseline Characteristics

The current study included 3349 participants (52.22% men, mean age: 60.80 years).  Table 1 describes the demographic and clinical characteristics at baseline of the analytical population in accordance with TyG index quintiles. Differences between groups were statistically significant for most variables (all *p* < 0.05) except for sex, BMI, SBP, hypertension, CVD, the medication of hypoglycemic agents, and lipid-lowering drugs. All biochemical variables at baseline between groups were statistically significant (all *p* < 0.05) as shown in Table 2.

### 3.2. HRs for Mortality

**Figure 1:**
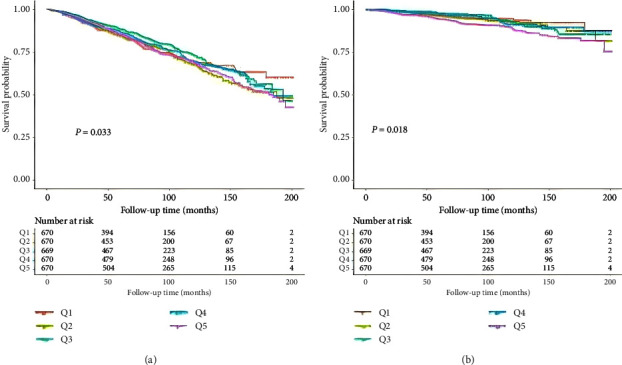
Kaplan–Meier survival curves for all-cause (a) and cardiovascular (b) mortality by TyG index. TyG, triglyceride-glucose index.

The median follow-up duration was 81.77 (SD: 49.44) months, during which 800 (23.89%) deaths due to all causes and 190 (5.6%) cardiovascular causes occurred. The cumulative survival probability stratified by the TyG index groups was significantly different in all-cause mortality (Log-rank *p*=0.033) and cardiovascular mortality (Log-rank *p*=0.018), according to Kaplan–Meier survival curves (Figure 1).

Table 3 shows the estimated HRs (and 95% CIs) for mortality from all causes and cardiovascular causes related to the TyG index. In the fully-adjusted model, per SD increment of TyG index were associated with increased risks of all-cause mortality (HR, 1.20; 95% CI, 1.05–1.38) and cardiovascular mortality (HR, 1.61; 95% CI, 1.23–2.11) when regarding TyG index as a continuous variable. In Model 3, participants in the top quintile (TyG index ≥ 9.83) had increased risks of all-cause mortality (HR, 1.38; 95% CI, 1.04–1.48) and cardiovascular mortality (HR, 2.43; 95% CI, 1.32–4.45) compared to the bottom quintile (TyG index ≤ 8.63) when regarding TyG index as a categorical variable.

**Figure 2:**
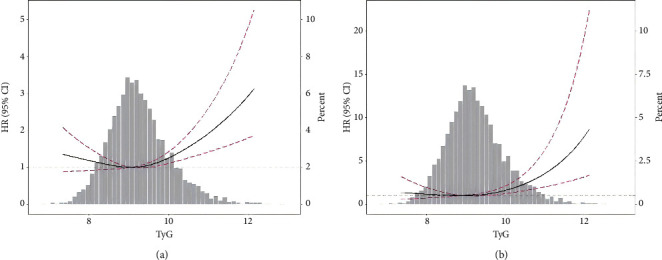
Spline analyses of the associations of TyG analyses with all-cause (a) and cardiovascular (b) mortality. Models were adjusted for age, gender, race, smoking, body mass index, systolic blood pressure, estimated glomerular filtration rate, total cholesterol, high-density lipoprotein cholesterol, cardiovascular disease, diabetes, hypertension, antihypertensive drugs, hypoglycemic agents, lipid-lowering drugs, and antiplatelet drugs. TyG, triglyceride-glucose index.

Restricted cubic spline models detected nonlinear associations of TyG index with all-cause mortality and cardiovascular mortality (Figure 2), revealing a J-shaped association between TyG index and all-cause mortality and a reversed L-shaped association between TyG index and cardiovascular mortality. Two piecewise linear regression models indicated that the all-cause and cardiovascular mortality thresholds were 9.32 and 9.37, respectively (Table 4). TyG index elevation over the threshold points was associated with increased risks of all-cause mortality (per SD increment, HR, 1.52; 95% CI, 1.27–1.82) and cardiovascular mortality (per SD increment, HR, 2.17; 95% CI, 1.54–3.04).

### 3.3. Subgroup Analyses

Analysis of subgroups (age, gender, BMI, and race) showed that TyG index above the threshold points resulted in higher risks for all-cause mortality and cardiovascular mortality with consistency. The subgroup analysis conducted for total energy had comparable results (Table S5). However, it showed no significant difference when TyG index was below the threshold points except for age stratification (Table 5). When TyG index < 9.32, for each 1-SD increment in TyG index, all-cause mortality decreased in participants aged ≥ 65 years (HR, 0.66; 95% CI, 0.50–0.86), but increased in participants aged < 65 years (HR, 1.55; 95% CI, 1.20–1.99).

### 3.4. Sensitivity Analyses

We utilized sensitivity analyses to assess the stability of the relations. The association between TyG index and the risk of all-cause mortality and cardiovascular mortality was not materially altered after excluding 760 participants who had cancer, heart failure, and renal failure at baseline. In the fully-adjusted model, per SD increment of TyG index were associated with increased risks of all-cause mortality (HR, 1.23; 95% CI, 1.08–1.40) and cardiovascular mortality (HR, 1.58; 95% CI, 1.22–2.05) when regarding TyG index as a continuous variable. In Model 3, participants in the top quintile (TyG index ≥ 9.83) had increased risks of all-cause mortality (HR, 1.99; 95% CI, 1.37–2.89) and cardiovascular mortality (HR, 4.01; 95% CI, 1.79–8.96) compared to the bottom quintile (TyG index ≤ 8.63) when regarding TyG index as a categorical variable (Table S1). Two piecewise linear regression models for sensitivity analysis showed that the threshold points of all-cause mortality and cardiovascular mortality were 9.34 and 9.38, respectively (Table S2), which were practically consistent with the primary analysis. TyG index elevation over the threshold points was associated with increased risks of all-cause mortality (per SD increment, HR, 1.47; 95% CI, 1.23–1.72) and cardiovascular mortality (per SD increment, HR, 3.21; 95% CI, 1.62–4.80). After adding the total energy variable into the analysis in Model 4 (Table S3), per SD increment of TyG index were associated with increased risks of all-cause mortality (HR, 1.21; 95% CI, 1.06–1.38) and cardiovascular mortality (HR, 1.62; 95% CI, 1.24–2.12) when regarding TyG index as a continuous variable. Participants in the top quintile (TyG index ≥ 9.83) had increased risks of all-cause mortality (HR, 1.39; 95% CI, 1.05–1.85) and cardiovascular mortality (HR, 2.44; 95% CI, 1.33–4.47) compared to the bottom quintile (TyG index ≤ 8.63) when regarding TyG index as a categorical variable. Two piecewise linear regression models for sensitivity analysis showed that the threshold points of all-cause mortality and cardiovascular mortality were 9.32 and 9.37, respectively (Table S4). TyG index elevation over the threshold points was associated with increased risks of all-cause mortality (per SD increment, HR, 1.53; 95% CI, 1.27–1.84) and cardiovascular mortality (per SD increment, HR, 2.29; 95% CI, 1.62–3.23).

## 4. Discussion

In this retrospective analysis, we discovered a nonlinear dose-response association between TyG index and mortality from all causes and cardiovascular causes in DM patients. TyG index was found to be an independent predictor of all-cause mortality and cardiovascular mortality after adjusting for potential confounding factors. Furthermore, we also identified the optimal threshold points were 9.32 and 9.37 for predicting all-cause mortality and cardiovascular mortality, respectively. Above the threshold points, the TyG index was found to be positively associated with higher all-cause mortality and cardiovascular mortality.

Previous literature has verified positive relationships between TyG index and mortality in different populations, which were consistent with our findings. TyG index in the upper tertile was associated with an increased risk of all-cause mortality in patients with DM and acute coronary syndrome after percutaneous coronary intervention therapy, compared to the tertile in the lower [24]. Consistently, TyG index and all-cause mortality were observed to be positively correlated in patients with chronic coronary syndrome or acute ischemic stroke [25, 26]. An observational prospective cohort research from Korea found that cardiovascular mortality was significantly more prevalent in the higher TyG group than in the equivalent lower TyG group among people who were metabolically unhealthy obese [27]. Individuals with both chronic heart failure and DM exhibited a positive correlation between TyG index and cardiovascular mortality [28]. In a retrospective study, the study of TyG index was divided into tertiles among patients in heart failure with reduced ejection fraction [29]. After controlling for confounders, the study found that individuals in tertile two and tertile three of the TyG index had a 2.24 times and 3.88 times higher risk of long-term mortality, respectively, compared to those in the lowest tertile [29]. Additionally, our analyses demonstrated the prediction of mortality risk using threshold points of the TyG index. Zhao et al. determined that TyG index of 9.18 was the best possible critical value for predicting the primary outcomes including non-fatal myocardial infarction, ischemia-driven revascularization along with all-cause death in patients with DM and non-ST-segment elevation acute coronary syndrome [30]. They also discovered that in multivariate Cox proportional hazards analysis, a one-unit elevation in TyG index was independently attributed to 3.2 times higher risks of the primary endpoints after controlling for confounding variables [30]. In our research of diabetic patients, however, a one-unit increment of TyG index, 1.20- and 1.61-times higher risks elevated in all-cause mortality and cardiovascular mortality, respectively. In another study involving 2531 consecutive patients with DM and acute coronary syndrome showed 9.32 was the cut-off point for predicting adverse cardiovascular events, which was approaching to the threshold values we discovered [31]. Consistent with the previous studies, our study demonstrated that both all-cause mortality and cardiovascular mortality had optimal threshold values for prediction. A study found that the cut-off point of TyG index was 9.68, and the risk of developing MACE was higher when TyG index ≥ 9.68 [8]. The cut-off point was different in our investigation, as their study focused on a population with a high risk of CVD and utilized MACE as the endpoint of the study's objective [8]. Biter et al. explored the efficacy of the TyG index in nondiabetic coronavirus disease 2019 (COVID-19) patients with myocardial injury [32]. Patients with the TyG index greater than 4.92 and nondiabetic COVID-19 who sustained myocardial infarction were found to have an elevated risk of in-hospital mortality [32]. The cut-off value of this study was obviously lower than the previous studies, which may be caused by the altered glucose and lipid metabolism as well as certain specific inflammatory factors [32]. Nevertheless, not all studies have found significantly nonlinear associations. Several studies demonstrated TyG index was linearly associated with ischemic stroke in general populations [33, 34]. Vega's study revealed that there was no significant relationship between TyG index and mortality risk in men after adjusting confounders [35]. Additionally, a meta-analysis also found no meaningful correlation between a higher TyG index and the increased risks of all-cause mortality and cardiovascular mortality in the general population [36]. These disparities may have been caused by differences in study populations, participant inclusion and exclusion criteria, event definitions, or study methodology.

In the current study, the subgroup analysis showed when TyG index < 9.32, for every one-SD increase in TyG index, all-cause mortality decreased in participants aged ≥ 65 years, but increased in participants aged < 65 years. A prospective cohort study included 359,645 Korean adults and demonstrated U-shaped associations between FBG and mortality in diabetic patients [37]. The study observed that in middle-aged and elderly DM patients (aged 45–64 years and ≥ 65 years), FBG below 100 mg/dL (< 5.6 mmol/L) at a mildly to moderately low concentration was associated with relatively increased risks of all-cause mortality [37]. A recent study from Chinese Longitudinal Healthy Longevity Survey (CLHLS) revealed older adults with an average age of 84.84 years should maintain a TG level no less than 1.66 mmol/L [38]. Compared to the lowest TG quartile (< 0.84 mmol/L), the highest TG quartile (≥ 1.66 mmol/L) had 49% lower risks of all-cause mortality [38]. Moreover, using univariable and multivariable analyses, a cross-sectional study of DM patients with a mean age of 58.31 years revealed that low levels of TG were associated with increased CVD risks among patients with a long duration of DM. However, among patients with a shorter DM duration, elevated TG levels were similarly correlated with increased CVD risks [39]. Diabetes and insulin resistance were more prevalent in the elderly than in the young, and they were associated with frailty, a type of geriatric disease that was physiologically vulnerable to stressors and was associated with adverse outcomes such as disability and mortality [40]. Other characteristics, such as a person's body composition, including their level of nutrition condition and muscle mass, may be age-related mortality predictors [41]. In this regard, our study provided additional evidence of an age-related correlation between TyG index and mortality from all causes in diabetic patients.

Possible mechanisms for these results were described below. Firstly, prior researches proved that cumulative long-term TyG index exposure was closely associated with the CVD risks, which contributed to increased mortality [42, 43]. Additionally, TyG index, as a substitution for IR, was associated with cardiovascular events and organ damage through oxidative stress, inflammation, prothrombotic events, and endothelial dysfunction, which may have an effect on individual life span [36, 43]. Finally, TyG index may have a major impact on modulating insulin signaling pathways, which aggravated IR in diabetic patients, according to the perspective of molecular processes [2, 36].

Several limitations in our investigation remained to be discussed. First of all, we did not conduct verification test concerning consistency of results between TyG index and HOMA-IR or HEC. Secondly, our investigation did not detect the dynamic change of the TyG index, which may provide additional information. Thirdly, lifestyle factors and behavior patterns such as physical activity and daily diet were not included in the analysis, which could bring about bias. Lastly, because our study was limited to diabetic patients in the United States, it might not be appropriate enough proxy for other demographics or geographical regions.

## 5. Conclusions

We demonstrated that the elevation of TyG index was associated with increased risk of mortality from all causes and cardiovascular causes among diabetic patients, suggesting TyG index may be a valuable clinical predictor of all-cause mortality and cardiovascular mortality in diabetic patients.

## Figures and Tables

**Table 1:** Demographic and clinical characteristics according to TyG index in quintiles at baseline.

	Total	TyG index					**p** value
		Q1 (≤ 8.63)	Q2 (8.63–9.00)	Q3 (9.01–9.35)	Q4 (9.36–9.82)	Q5 (≥ 9.83)	
Number	3349	670	670	669	670	670	
Age, years	60.80 ± 14.53	61.23 ± 15.09	61.98 ± 14.51	61.73 ± 13.86	61.24 ± 14.42	57.80 ± 14.37	<0.001
Sex, *n* (%)							0.700
Male	1749 (52.22)	346 (51.64)	337 (50.30)	348 (52.02)	356 (53.13)	362 (54.03)	
Female	1600 (47.78)	324 (48.36)	333 (49.70)	321 (47.98)	314 (46.87)	308 (45.97)	
Race, *n* (%)							<0.001
Non-white	1993 (59.51)	439 (65.52)	395 (58.96)	380 (56.80)	368 (54.93)	411 (61.34)	
White	1356 (40.49)	231 (34.48)	275 (41.04)	289 (43.20)	302 (45.07)	259 (38.66)	
Smoking, *n* (%)							0.003
No	1584 (47.74)	343 (51.89)	346 (52.11)	297 (44.53)	297 (44.66)	301 (45.54)	
Yes	1734 (52.26)	318 (48.11)	318 (47.89)	370 (55.47)	368 (55.34)	360 (54.46)	
Body mass index, kg/m^2^	31.86 ± 7.35	31.28 ± 8.05	31.82 ± 7.80	32.12 ± 7.23	32.10 ± 6.56	31.97 ± 7.00	0.218
Systolic blood pressure, mmHg	132.51 ± 20.96	131.83 ± 20.94	131.01 ± 20.60	133.22 ± 21.55	132.96 ± 20.72	133.54 ± 20.96	0.165
Diastolic blood pressure, mmHg	68.72 ± 15.19	67.10 ± 16.27	67.84 ± 14.89	69.23 ± 14.50	69.52 ± 13.92	69.90 ± 16.06	0.003
Comorbidities, *n* (%)							
Hypertension							0.068
No	752 (22.47)	134 (20.03)	145 (21.64)	148 (22.16)	148 (22.09)	177 (26.46)	
Yes	2594 (77.53)	535 (79.97)	525 (78.36)	520 (77.84)	522 (77.91)	492 (73.54)	
Cardiovascular disease							0.166
No	2572 (77.45)	516 (78.06)	516 (77.59)	528 (79.16)	493 (73.91)	519 (78.52)	
Yes	749 (22.55)	145 (21.94)	149 (22.41)	139 (20.84)	174 (26.09)	142 (21.48)	
Treatment, *n* (%)							
Antihypertensive drugs							0.024
No	1384 (41.33)	249 (37.16)	283 (42.24)	272 (40.66)	272 (40.60)	308 (45.97)	
Yes	1965 (58.67)	421 (62.84)	387 (57.76)	397 (59.34)	398 (59.40)	362 (54.03)	
Hypoglycemic agents							0.067
No	1622 (48.43)	340 (50.75)	342 (51.04)	327 (48.88)	318 (47.46)	295 (44.03)	
Yes	1727 (51.57)	330 (49.25)	328 (48.96)	342 (51.12)	352 (52.54)	375 (55.97)	
Lipid-lowering drugs							0.082
No	2192 (65.45)	419 (62.54)	448 (66.87)	437 (65.32)	425 (63.43)	463 (69.10)	
Yes	1157 (34.55)	251 (37.46)	222 (33.13)	232 (34.68)	245 (36.57)	207 (30.90)	
Antiplatelet drugs							0.037
No	3176 (94.83)	643 (95.97)	636 (94.93)	622 (92.97)	630 (94.03)	645 (96.27)	
Yes	173 (5.17)	27 (4.03)	34 (5.07)	47 (7.03)	40 (5.97)	25 (3.73)	
Outcomes, *n* (%)							
Cardiovascular disease mortality							<0.001
No	3159 (94.33)	644 (96.12)	635 (94.78)	637 (95.22)	636 (94.93)	607 (90.60)	
Yes	190 (5.67)	26 (3.88)	35 (5.22)	32 (4.78)	34 (5.07)	63 (9.40)	
All-cause mortality							<0.001
No	2549 (76.11)	537 (80.15)	507 (75.67)	526 (78.62)	504 (75.22)	475 (70.90)	
Yes	800 (23.89)	133 (19.85)	163 (24.33)	143 (21.38)	166 (24.78)	195 (29.10)	

*Note:* Continuous variables were summarized as mean (SD) or medians (quartile interval) and categorical variables were displayed as percentage (%).

Abbreviations: Q, quintiles; TyG, triglyceride-glucose.

**Table 2:** Biochemical variables according to TyG index in quintiles at baseline.

	Total	TyG index					**p** value
		Q1 (≤ 8.63)	Q2 (8.63–9.00)	Q3 (9.01–9.35)	Q4 (9.36–9.82)	Q5 (≥ 9.83)	
eGFR, mg/min/1.73 m^2^	81.01 ± 29.71	80.63 ± 28.80	78.97 ± 29.71	78.82 ± 26.83	80.86 ± 29.75	85.78 ± 32.70	<0.001
Total cholesterol, mg/dL	192.49 ± 46.31	172.64 ± 38.63	180.91 ± 38.43	192.65 ± 40.35	196.39 ± 42.95	219.86 ± 54.78	<0.001
HDL cholesterol, mg/dL	48.93 ± 14.40	57.70 ± 17.03	51.71 ± 12.92	49.23 ± 12.35	45.26 ± 11.60	40.75 ± 11.37	<0.001
LDL cholesterol, mg/dL	109.08 ± 37.12	99.80 ± 32.87	106.60 ± 34.81	113.87 ± 36.99	112.56 ± 39.24	114.08 ± 40.37	<0.001
Triglycerides, mg/dL	179.30 ± 181.73	74.49 ± 22.45	110.13 ± 26.38	146.71 ± 35.71	192.73 ± 52.91	372.39 ± 325.01	<0.001
Fasting blood glucose, mg/dL	154.76 ± 64.54	113.76 ± 27.31	131.42 ± 31.15	140.81 ± 37.88	161.39 ± 51.75	226.41 ± 86.08	<0.001
Glycohemoglobin, %	7.14 ± 1.76	6.45 ± 1.14	6.57 ± 1.10	6.76 ± 1.23	7.28 ± 1.75	8.67 ± 2.26	<0.001
TyG index	9.26 ± 0.78	8.28 ± 0.30	8.83 ± 0.11	9.18 ± 0.10	9.57 ± 0.14	10.42 ± 0.54	<0.001

*Note:* Continuous variables were summarized as mean (SD) or medians (quartile interval) and categorical variables were displayed as percentage (%).

Abbreviations: HDL, high-density lipoprotein; LDL, low-density lipoprotein; *n*, number; Q, quintiles; TyG, triglyceride-glucose.

**Table 3:** Multivariate cox regression analysis of TyG index with cause-specific mortality.

	Event rate/1000 person-years	Model 1 HR (95% CI), **p** value	Model 2 HR (95% CI), **p** value	Model 3 HR (95% CI), **p** value
All-cause mortality				
TyG index (per SD increase)	35.06	0.99 (0.90, 1.08) 0.7771	1.11 (1.01, 1.22) 0.0388	1.20 (1.05, 1.38) 0.0071
TyG index group				
Q1 (≤8.63)	34.11	1.0	1.0	1.0
Q2 (8.63–-9.00)	37.31	1.07 (0.85, 1.35) 0.5362	0.99 (0.79, 1.25) 0.9354	1.01 (0.78, 1.29) 0.9630
Q3 (9.01–-9.35)	30.65	0.87 (0.69, 1.10) 0.2475	0.83 (0.66, 1.06) 0.1346	0.89 (0.68, 1.15) 0.3771
Q4 (9.36–-9.82)	34.52	0.97 (0.77, 1.22) 0.7881	0.91 (0.72, 1.15) 0.4233	0.94 (0.72, 1.23) 0.6771
Q5 (≥9.83)	38.41	1.06 (0.85, 1.33) 0.5771	1.25 (1.00, 1.56) 0.0491	1.38 (1.04, 1.84) 0.0262
P for trend		0.847	0.102	0.070
Cardiovascular mortality				
TyG index (per SD increase)	8.33	1.25 (1.05, 1.48) 0.0113	1.50 (1.24, 1.82) < 0.0001	1.61 (1.23, 2.11) 0.0005
TyG index group				
Q1 (≤8.63)	6.67	1.0	1.0	1.0
Q2 (8.63–-9.00)	8.01	1.18 (0.71, 1.96) 0.5205	1.09 (0.66, 1.81) 0.7362	1.18 (0.67, 2.10) 0.5601
Q3 (9.01–-9.35)	6.86	1.00 (0.59, 1.67) 0.9878	0.97 (0.58, 1.63) 0.9100	1.14 (0.64, 2.03) 0.6658
Q4 (9.36–-9.82)	7.07	1.01 (0.61, 1.69) 0.9552	0.96 (0.57, 1.60) 0.8749	0.94 (0.51, 1.75) 0.8534
Q5 (≥9.83)	12.41	1.75 (1.11, 2.77) 0.0168	2.14 (1.35, 3.39) 0.0012	2.43 (1.32, 4.45) 0.0041
P for trend		0.022	0.002	0.013

*Note:* Model 1 adjusted for none; Model 2 adjusted for age, gender, and race; Model 3 adjusted for age, gender, race, smoking, body mass index, systolic blood pressure, estimated glomerular filtration rate, total cholesterol, high-density lipoprotein cholesterol, comorbidities (cardiovascular disease and hypertension), and medicine use (antihypertensive drugs, hypoglycemic agents, lipid-lowering drugs, and antiplatelet drugs).

Abbreviations: CI, confidence interval; HR, hazard ratio; Q, quintiles; TyG, triglyceride-glucose.

**Table 4:** The results of two-piecewise linear regression model between TyG index and cause-specific mortality.

	All-cause mortality HR (95% CI), **p** value	Cardiovascular disease mortality HR (95% CI), **p** value
Cut-off value	9.32	9.37
< Cut-off value	0.85 (0.68, 1.08) 0.1802	0.92 (0.57, 1.50) 0.7491
≥ Cut-off value	1.52 (1.27, 1.82) < 0.0001	2.17 (1.54, 3.04) < 0.0001
*P* For log likelihood ratio test	<0.001	0.010

*Note:* The two-piecewise linear regression models were adjusted for age, gender, race, smoking, body mass index, systolic blood pressure, estimated glomerular filtration rate, total cholesterol, high-density lipoprotein cholesterol, comorbidities (cardiovascular disease and hypertension), and medicine use (antihypertensive drugs, hypoglycemic agents, lipid-lowering drugs, and antiplatelet drugs).

Abbreviations: CI, confidence interval; HR, hazard ratio; TyG, triglyceride-glucose.

**Table 5:** Subgroups analysis.

Cut-off value, mmol/L	*N*	All-cause mortality HR (95% CI), **p** value		P for log likelihood ratio test	Cardiovascular disease mortality HR (95% CI), **p** value		P for log likelihood ratio test
		< 9.32	≥ 9.32		< 9.37	≥ 9.37	
Age							
≥ 65	1357	0.66 (0.50, 0.86) 0.0019	1.55 (1.20, 1.99) 0.0008	<0.001	0.78 (0.44, 1.36) 0.3753	2.26 (1.39, 3.70) 0.0011	0.014
< 65	1706	1.69 (1.04, 2.74) 0.0329	1.32 (1.00, 1.75) 0.0465	0.419	1.35 (0.51, 3.62) 0.5451	1.74 (1.04, 2.92) 0.0345	0.685
Gender							
Male	1599	0.98 (0.72, 1.33) 0.8947	1.47 (1.15, 1.87) 0.0018	0.065	1.24 (0.64, 2.40) 0.5257	2.15 (1.42, 3.27) 0.0003	0.198
Female	1464	0.72 (0.50, 1.02) 0.0632	1.64 (1.23, 2.17) 0.0006	0.002	0.70 (0.33, 1.49) 0.3551	2.13 (1.16, 3.93) 0.0154	0.046
Race							
Non-white	1823	0.85 (0.62, 1.16) 0.3034	1.56 (1.21, 2.01) 0.0006	0.008	0.76 (0.40, 1.43) 0.3919	2.60 (1.66, 4.08) < 0.0001	0.005
White	1240	0.84 (0.60, 1.19) 0.3282	1.47 (1.13, 1.92) 0.0039	0.021	1.06 (0.50, 2.26) 0.8758	1.77 (1.02, 3.07) 0.0429	0.331
Body mass index, kg/m^2^							
< 25	462	0.65 (0.39, 1.06) 0.0867	2.87 (1.83, 4.49) < 0.0001	<0.001	0.55 (0.20, 1.46) 0.2274	4.69 (2.17, 10.11) < 0.0001	0.003
≥ 25	2601	0.87 (0.67, 1.14) 0.3116	1.38 (1.13, 1.70) 0.0021	0.016	1.01 (0.57, 1.80) 0.9597	1.93 (1.29, 2.89) 0.0015	0.103

*Note:* When analyzing a subgroup variable, age, gender, race, smoking, body mass index, systolic blood pressure, estimated glomerular filtration rate, total cholesterol, high-density lipoprotein cholesterol, comorbidities (cardiovascular disease and hypertension), and medicine use (antihypertensive drugs, hypoglycemic agents, lipid-lowering drugs, and antiplatelet drugs) were all adjusted except the variable itself.

Abbreviations: CI, confidence interval; TyG, triglyceride-glucose.

## Data Availability

All data relevant to the study are included within the article.

## Supporting Information

Figure S1: 3349 subjects with DM were included as the analytic population. Table S1: Sensitivity analysis of the Cox regression models by excluding participants with heart failure, renal failure (eGFR < 30 mg/min/1.73 m^2^), and/or cancer at baseline. Table S2: Sensitivity analysis of the two-piecewise linear regression models by excluding participants with heart failure, renal failure (eGFR < 30 mg/min/1.73 m^2^), and/or cancer at baseline. Table S3: Sensitivity analysis of the Cox regression models by adding total energy variable at baseline. Table S4: Sensitivity analysis of the two-piecewise linear regression models by adding total energy variable at baseline. Table S5: Subgroups analysis for total energy grouped by median value.
