# Supplementary material for: Predictive Value of Triglyceride-Glucose Index for All-Cause and Cardiovascular Mortality in Patients With Diabetes Mellitus: A Retrospective Study: TyG Index and Mortality in Diabetes
Source: Int J Endocrinol. 2024 Oct 23;2024:6417205. doi: 10.1155/2024/6417205 (PMC11524704; doi:10.1155/2024/6417205)
Supplement: Supporting Information — Additional supporting information can be found online in the Supporting Information section. [file 6417205.f1.zip › Supplementary Tables (1).docx]

**Table S1** Sensitivity analysis of the Cox regression models by excluding participants with heart failure, renal failure, and/or cancer at baseline.

|  |  | **Event rate/ 1000 person-years** | | **Model 1**  **HR (95%CI), *P*-value** | **Model 2**  **HR (95%CI), *P*-value** | | **Model 3**  **HR (95%CI), *P*-value** | |
| --- | --- | --- | --- | --- | --- | --- | --- | --- |
| All-cause mortality | | | | | | | |  |
| TyG index (per SD increase) |  | 26.67 | | 1.03 (0.95, 1.13) 0.485 | 1.12 (1.02, 1.23) 0.021 | | 1.23 (1.08, 1.40) 0.001 | |
| TyG index group |  |  | |  |  | |  | |
| Q1 (≤ 8.63) |  | 23.54 | | 1.0 | 1.0 | | 1.0 | |
| Q2 (8.64-9.01) |  | 27.26 | | 1.14 (0.84, 1.54) 0.400 | 1.06 (0.79, 1.44) 0.692 | | 1.20 (0.87, 1.66) 0.274 | |
| Q3 (9.02-9.37) |  | 22.58 | | 0.93 (0.68, 1.27) 0.649 | 0.93 (0.68, 1.26) 0.627 | | 1.06 (0.75, 1.49) 0.752 | |
| Q4 (9.37-9.87) |  | 27.85 | 1.12 (0.84, 1.51) 0.442 | | 1.05 (0.78, 1.41) 0.731 | 1.24 (0.88, 1.74) 0.226 | |  |
| Q5 (≥9.88) |  | 31.26 | 1.25 (0.94, 1.66) 0.128 | | 1.44 (1.08, 1.93) 0.012 | 1.99 (1.37, 2.89) <0.001 | |  |
| P for trend |  |  | 0.117 | | 0.087 | 0.059 | |  |
| Cardiovascular mortality | | | | | | | |  |
| TyG index (per SD increase) |  | 6.22 | 1.26 (1.07, 1.48) 0.007 | | 1.44 (1.20, 1.72) < 0.001 | 1.58 (1.22, 2.05) <0.001 | |  |
| TyG index group |  |  |  | |  |  | |  |
| Q1 (≤ 8.63) |  | 4.03 | 1.0 | | 1.0 | 1.0 | |  |
| Q2 (8.64-9.01) |  | 6.18 | 1.51 (0.76, 2.99) 0.241 | | 1.42 (0.72, 2.83) 0.313 | 1.70 (0.81, 3.60) 0.163 | |  |
| Q3 (9.02-9.37) |  | 5.17 | 1.25 (0.62, 2.53) 0.541 | | 1.25 (0.62, 2.52) 0.542 | 1.49 (0.69, 3.23) 0.315 | |  |
| Q4 (9.37-9.87) |  | 5.44 | 1.21 (0.60, 2.44) 0.593 | | 1.14 (0.56, 2.29) 0.723 | 1.45 (0.65, 3.24) 0.361 | |  |
| Q5 (≥9.88) |  | 10.89 | 2.32 (1.24, 4.34) 0.009 | | 2.84 (1.51, 5.33) 0.001 | 4.01 (1.79, 8.96) <0.001 | |  |
| P for trend |  |  | 0.007 | | <0.001 | <0.001 | |  |

Model 1 adjusted for none; Model 2 adjusted for age, gender, and race; Model 3 adjusted for age, gender, race, smoking, body mass index, systolic blood pressure, estimated glomerular filtration rate, total cholesterol, high density lipoprotein cholesterol, cardiovascular disease, hypertension, antihypertensive drugs, hypoglycemic agents, lipid-lowering drugs, and antiplatelet drugs.

Abbreviations: TyG, triglyceride-glucose; HR, hazard ratio; CI, confidence interval; Q, quintiles.

**Table S2** Sensitivity analysis of the two-piecewise linear regression models by excluding participants with heart failure, renal failure, and/or cancer at baseline.

|  | **All-cause mortality**  **HR (95% CI),** ***P*-value** | **Cardiovascular disease mortality**  **HR (95% CI), *P*-value** |
| --- | --- | --- |
| Cutoff value | 9.34 | 9.38 |
| <Cut-off value | 0.95 (0.78, 1.10) 0.221 | 0.89 (0.48, 1.31) 0.628 |
| ≥Cut-off value | 1.47 (1.23, 1.72) <0.001 | 3.21 (1.62, 4.80) <0.001 |
| *P* for log likelihood ratio test | <0.001 | <0.001 |

The two-piecewise linear regression models were adjusted for age, gender, race, smoking, body mass index, systolic blood pressure, estimated glomerular filtration rate, total cholesterol, high density lipoprotein cholesterol, cardiovascular disease, hypertension, antihypertensive drugs, hypoglycemic agents, lipid-lowering drugs, and antiplatelet drugs.

Abbreviations: TyG, triglyceride-glucose; HR, hazard ratio; CI, confidence interval.

|  |  | **Event rate/ 1000 person-years** | **Model 1**  **HR (95%CI), *P*-value** | **Model 2**  **HR (95%CI), *P*-value** | **Model 3**  **HR (95%CI), *P*-value** | **Model 4**  **HR (95%CI), *P*-value** |
| --- | --- | --- | --- | --- | --- | --- |
| All-cause mortality | | | | | |  |
| TyG index (per SD increase) |  | 35.06 | 0.99 (0.90, 1.08) 0.7771 | 1.11 (1.01, 1.22) 0.0388 | 1.20 (1.05, 1.38) 0.0071 | 1.21 (1.06-1.38) 0.0057 |
| TyG index group |  |  |  |  |  |  |
| Q1 (≤ 8.63) |  | 34.11 | 1.0 | 1.0 | 1.0 | 1.0 |
| Q2 (8.64-9.01) |  | 37.31 | 1.07 (0.85, 1.35) 0.5362 | 0.99 (0.79, 1.25) 0.9354 | 1.01 (0.78, 1.29) 0.9630 | 1.01 (0.78-1.30) 0.9464 |
| Q3 (9.02-9.37) |  | 30.65 | 0.87 (0.69, 1.10) 0.2475 | 0.83 (0.66, 1.06) 0.1346 | 0.89 (0.68, 1.15) 0.3771 | 0.89 (0.69-1.16) 0.3865 |
| Q4 (9.37-9.87) |  | 34.52 | 0.97 (0.77, 1.22) 0.7881 | 0.91 (0.72, 1.15) 0.4233 | 0.94 (0.72, 1.23) 0.6771 | 0.95 (0.73-1.24) 0.7006 |
| Q5 (≥9.88) |  | 38.41 | 1.06 (0.85, 1.33) 0.5771 | 1.25 (1.00, 1.56) 0.0491 | 1.38 (1.04, 1.84) 0.0262 | 1.39 (1.05-1.85) 0.0231 |
| P for trend |  |  | 0.847 | 0.102 | 0.070 | 0.063 |
| Cardiovascular mortality | |  | | |  |  |
| TyG index (per SD increase) |  | 8.33 | 1.25 (1.05, 1.48) 0.0113 | 1.50 (1.24, 1.82) <0.0001 | 1.61 (1.23, 2.11) 0.0005 | 1.62 (1.24-2.12) 0.0005 |
| TyG index group |  |  |  |  |  |  |
| Q1 (≤ 8.63) |  | 6.67 | 1.0 | 1.0 | 1.0 | 1.0 |
| Q2 (8.64-9.01) |  | 8.01 | 1.18 (0.71, 1.96) 0.5205 | 1.09 (0.66, 1.81) 0.7362 | 1.18 (0.67, 2.10) 0.5601 | 1.19 (0.67-2.10) |
| Q3 (9.02-9.37) |  | 6.86 | 1.00 (0.59, 1.67) 0.9878 | 0.97 (0.58, 1.63) 0.9100 | 1.14 (0.64, 2.03) 0.6658 | 1.14 (0.64-2.04) |
| Q4 (9.37-9.87) |  | 7.07 | 1.01 (0.61, 1.69) 0.9552 | 0.96 (0.57, 1.60) 0.8749 | 0.94 (0.51, 1.75) 0.8534 | 0.94 (0.51-1.76) |
| Q5 (≥9.88) |  | 12.41 | 1.75 (1.11, 2.77) 0.0168 | 2.14 (1.35, 3.39) 0.0012 | 2.43 (1.32, 4.45) 0.0041 | 2.44 (1.33-4.47) |
| P for trend |  |  | 0.022 | 0.002 | 0.013 | 0.012 |

**Table S3** Sensitivity analysis of the Cox regression models by adding total energy variable at baseline.

Model 1 adjusted for none; Model 2 adjusted for age, gender, and race; Model 3 adjusted for age, gender, race, smoking, body mass index, systolic blood pressure, estimated glomerular filtration rate, total cholesterol, high density lipoprotein cholesterol, cardiovascular disease, hypertension, antihypertensive drugs, hypoglycemic agents, lipid-lowering drugs, and antiplatelet drugs; Model 4 adjusted for Model 3 + total energy.

Abbreviations: TyG, triglyceride-glucose; HR, hazard ratio; CI, confidence interval; Q, quintiles.

**Table S4** Sensitivity analysis of the two-piecewise linear regression models by adding total energy variable at baseline..

|  | **All-cause mortality**  **HR (95% CI), *P*-value** | **Cardiovascular disease mortality**  **HR (95% CI), *P*-value** |
| --- | --- | --- |
| Cutoff value | 9.32 | 9.37 |
| <Cut-off value | 0.89 (0.70, 1.13) 0.333 | 0.87 (0.54, 1.42) 0.587 |
| ≥Cut-off value | 1.53 (1.27, 1.84) <0.001 | 2.29 (1.62, 3.23) <0.001 |
| *P* for log likelihood ratio test | 0.002 | 0.004 |

The two-piecewise linear regression models were adjusted for age, gender, race, smoking, body mass index, systolic blood pressure, estimated glomerular filtration rate, total cholesterol, high density lipoprotein cholesterol, total energy, cardiovascular disease, hypertension, antihypertensive drugs, hypoglycemic agents, lipid-lowering drugs, and antiplatelet drugs.

Abbreviations: TyG, triglyceride-glucose; HR, hazard ratio; CI, confidence interval.

**Table S5** Subgroups analysis for total energy grouped by median value.

|  | **N** | **All-cause mortality**  **HR (95% CI), *P*-value** | | ***P* for log likelihood ratio test** | **Cardiovascular disease mortality**  **HR (95% CI), *P*-value** | | ***P* for log**  **likelihood ratio test** |
| --- | --- | --- | --- | --- | --- | --- | --- |
| **Cutoff value, mmol/L** |  | **<9.32** | **≥9.32** |  | **<9.37** | **≥9.37** |  |
| Total energy |  |  |  |  |  |  |  |
| >=1719.5 | 1470 | 0.86 (0.60-1.22) 0.3977 | 1.49 (1.15-1.93) 0.0025 | 0.024 | 1.17 (0.54-2.55) 0.6949 | 2.49 (1.50-4.14) 0.0004 | 0.202 |
| <1719.5 | 1464 | 0.84 (0.62-1.15) 0.2862 | 1.60 (1.22-2.11) 0.0008 | 0.008 | 0.81 (0.43-1.54) 0.5236 | 2.32 (1.35-3.97) 0.0022 | 0.031 |

When analyzing a subgroup variable, age, gender, race, smoking, body mass index, systolic blood pressure, estimated glomerular filtration rate, total cholesterol, high density lipoprotein cholesterol, comorbidities (cardiovascular disease and hypertension), and medicine use (antihypertensive drugs, hypoglycemic agents, lipid-lowering drugs, and antiplatelet drugs), total energy were all adjusted except the variable itself.

Abbreviations: TyG, triglyceride-glucose; CI, confidence interval.
