# Supplementary figures and images for: Predictive Value of Triglyceride-Glucose Index for All-Cause and Cardiovascular Mortality in Patients With Diabetes Mellitus: A Retrospective Study: TyG Index and Mortality in Diabetes
Source: Int J Endocrinol. 2024 Oct 23;2024:6417205. doi: 10.1155/2024/6417205 (PMC11524704; doi:10.1155/2024/6417205)

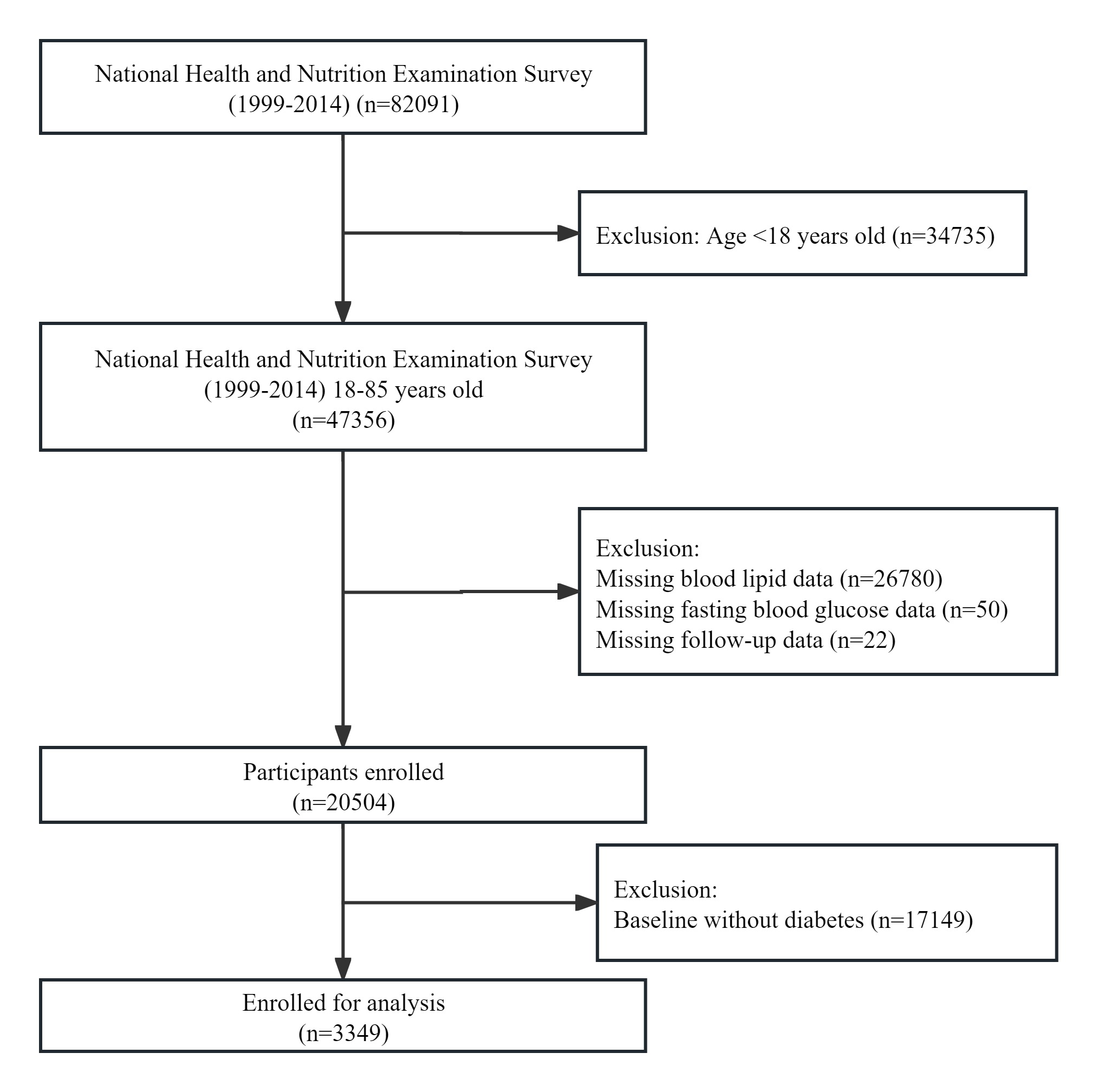

Supplement: Supporting Information — Additional supporting information can be found online in the Supporting Information section. [file 6417205.f1.zip › Figure S1 (2).jpg]
